# Higher risk of death from COVID-19 in low-income and non-White populations of São Paulo, Brazil

**DOI:** 10.1136/bmjgh-2021-004959

**Published:** 2021-04-29

**Authors:** Sabrina L Li, Rafael H M Pereira, Carlos A Prete Jr, Alexander E Zarebski, Lucas Emanuel, Pedro J H Alves, Pedro S Peixoto, Carlos K V Braga, Andreza Aruska de Souza Santos, William M de Souza, Rogerio J Barbosa, Lewis F Buss, Alfredo Mendrone, Cesar de Almeida-Neto, Suzete C Ferreira, Nanci A Salles, Izabel Marcilio, Chieh-Hsi Wu, Nelson Gouveia, Vitor H Nascimento, Ester C Sabino, Nuno R Faria, Jane P Messina

**Affiliations:** 1 School of Geography and the Environment, University of Oxford, Oxford, UK; 2 Institute of Applied Economic Research, Brasília, Brazil; 3 Department of Electronic Systems Engineering, University of São Paulo, São Paulo, Brazil; 4 Department of Zoology, University of Oxford, Oxford, UK; 5 Department of Applied Mathematics, Institute of Mathematics and Statistics, University of São Paulo, São Paulo, Brazil; 6 Oxford School of Global and Area Studies, Latin American Centre, University of Oxford, Oxford, UK; 7 Virology Research Center, Ribeirão Preto Medical School, University of São Paulo, Ribeirão Preto, Brazil; 8 Institute of Social and Political Studies (IESP), State University of Rio de Janeiro (UERJ), Rio de Janeiro, Brazil; 9 Departamento de Molestias Infecciosas e Parasitarias andInstituto de Medicina Tropical, Faculdade de Medicina da Universidade de São Paulo, São Paulo, Brazil; 10 Fundação Pró-Sangue Hemocentro de São Paulo, São Paulo, Brazil; 11 Disciplina de Ciências Médicas, Faculdade de Medicina da Universidade de São Paulo, São Paulo, Brazil; 12 Laboratory of Medical Investigation in Pathogenesis and Directed Therapy in Onco – Immuno – Hematology (LIM-31) HCFMUSP, University of São Paulo Medical School, São Paulo, Brazil; 13 Hospital das Clinicas da Faculdade de Medicina da Universidade de São Paulo, University of São Paulo, São Paulo, Brazil; 14 Mathematical Sciences, University of Southampton, Southampton, UK; 15 Department of Preventive Medicine, University of São Paulo Medical School, São Paulo, Brazil; 16 MRC Centre for Global Infectious Disease Analysis; and the Abdul Latif Jameel Institute for Disease and Emergency Analytics (J-IDEA), School of Public Health, Imperial College London, London, UK; 17 Oxford School of Global and Area Studies, University of Oxford, Oxford, UK

**Keywords:** geographic information systems, epidemiology, public health, cross-sectional survey, mathematical modelling

## Abstract

**Introduction:**

Little evidence exists on the differential health effects of COVID-19 on disadvantaged population groups. Here we characterise the differential risk of hospitalisation and death in São Paulo state, Brazil, and show how vulnerability to COVID-19 is shaped by socioeconomic inequalities.

**Methods:**

We conducted a cross-sectional study using hospitalised severe acute respiratory infections notified from March to August 2020 in the *Sistema de Monitoramento Inteligente de São Paulo* database. We examined the risk of hospitalisation and death by race and socioeconomic status using multiple data sets for individual-level and spatiotemporal analyses. We explained these inequalities according to differences in daily mobility from mobile phone data, teleworking behaviour and comorbidities.

**Results:**

Throughout the study period, patients living in the 40% poorest areas were more likely to die when compared with patients living in the 5% wealthiest areas (OR: 1.60, 95% CI 1.48 to 1.74) and were more likely to be hospitalised between April and July 2020 (OR: 1.08, 95% CI 1.04 to 1.12). Black and *Pardo* individuals were more likely to be hospitalised when compared with White individuals (OR: 1.41, 95% CI 1.37 to 1.46; OR: 1.26, 95% CI 1.23 to 1.28, respectively), and were more likely to die (OR: 1.13, 95% CI 1.07 to 1.19; 1.07, 95% CI 1.04 to 1.10, respectively) between April and July 2020. Once hospitalised, patients treated in public hospitals were more likely to die than patients in private hospitals (OR: 1.40%, 95% CI 1.34% to 1.46%). Black individuals and those with low education attainment were more likely to have one or more comorbidities, respectively (OR: 1.29, 95% CI 1.19 to 1.39; 1.36, 95% CI 1.27 to 1.45).

**Conclusions:**

Low-income and Black and *Pardo* communities are more likely to die with COVID-19. This is associated with differential access to quality healthcare, ability to self-isolate and the higher prevalence of comorbidities.

**Key questions**

**What is already known?**

- Black and *Pardo* (mixed ethnicity) Brazilians face higher risk of COVID-19 hospitalised death.
- Access to COVID-19 testing has been limited for low-income populations in São Paulo city.

**What are the new findings?**

- Individual and population-level risk of COVID-19 hospitalisation, death and adherence to non-pharmaceutical interventions vary by race and socioeconomic status.
- Low socioeconomic and/or Black and *Pardo* (Brazilians of mixed ethnic ancestries) communities have lower levels of social isolation and face higher risks of hospitalisation and death.

What do the new findings imply?

- The stark difference in COVID-19 mortality between public and private healthcare settings underscores the need for further investigation on the drivers of mortality in different hospital settings.
- While non-pharmaceutical interventions have been implemented in São Paulo and other states to slow down transmission, the effectiveness of these interventions among population groups varies with socioeconomic status.
- Healthcare workers, disadvantaged groups working in face-to-face occupations in crowded and segregated areas should be prioritised for vaccination.

## Introduction

The COVID-19 pandemic has amplified the effects of social inequalities on exposure and death in low socioeconomic groups,^1^ particularly in Brazil, where it has caused significant mortality.^2^ The prevalence of COVID-19 mortality is partially driven by pre-existing non-communicable diseases, which are socially clustered due to entrenched inequalities.^3^ These inequalities are shaped by the social determinants of health,^4^ which define a population’s health based on the environments where they ‘grow, live, work, and age’, from birth.^5^ Even when underlying health conditions are not present, the interactions of these social determinants disproportionately expose disadvantaged groups to COVID-19^4^ and other conditions that could induce adverse chronic health conditions.^6^ Furthermore, a review has found that disadvantaged groups are the most vulnerable to the psychosocial impacts of COVID-19,^7^ which can aggravate the severity of COVID-19.^4^

Several studies that have been conducted in the context of high-income countries have mostly focused on the USA,^8–10^ UK^11 12^ and European countries,^13 14^ which have consistently found that populations identified as non-White, of low socioeconomic status and those living in high poverty were associated with higher SARS-CoV-2 transmission and COVID-19 death. Few studies have addressed the uneven impact of COVID-19 by socioeconomic status and race in low and middle-income countries,^15^ in part because national surveillance systems seldom collect or report this information.^16^ In Brazil, higher risk of COVID-19 death has been found for Black and *Pardo* (mixed ethnicity) Brazilians, especially those who are identified as male with low socioeconomic status.^17 18^ Nonetheless, there is still little information on how the differential health outcomes of COVID-19 are shaped by broader social inequalities that determine the capacity to self-isolate and non-pharmaceutical interventions (NPIs).

It is paramount to understand the potential social drivers of COVID-19 morbidity and mortality, particularly in countries with high inequality such as Brazil.^19^ The first COVID-19 cases in Brazil were detected in São Paulo,^20^ the most populous state and home to diverse racial groups. In the Brazilian context of politically polarised public health responses,^21^ São Paulo has been severely affected by COVID-19^22^ and access to testing has been limited for low-income populations.^23^

We conducted a multiscale analysis to investigate the risk of hospitalisation and death from severe acute respiratory infections (SARI), predominantly caused by COVID-19,^23^ notified from March to August 2020, in the *Sistema de Monitoramento Inteligente de São Paulo* (SIMI-SP) database, for São Paulo state. We considered all SARI cases instead of only including patients who tested positive for COVID-19 to avoid the bias in access to SARS-CoV-2 testing towards higher socioeconomic classes in Brazil,^23^ which allows us to better capture the disproportionate impact of social inequities on racial and socioeconomic groups. We examined differential risk by race and socioeconomic status, by combining multiple high-resolution data from mobile phones, government census and population surveys conducted during the epidemic. We assessed potential drivers of these inequalities by evaluating local levels of self-isolation, access to teleworking and prevalence of comorbidities.

## Methods

### Data sources

#### SARI and patient information

Patient-level information on demographic characteristics, home address, hospitalisation and health outcomes was collected from the São Paulo State Health Secretariat SARI hospitalisations database (SIMI-SP).^24^ SARI can be caused by SARS-CoV-2 and is defined by the Brazilian Ministry of Health as influenza-like syndrome plus one of the following: dyspnoea, persistent chest pain or hypoxia. We excluded all SARI cases that were confirmed to be caused by other respiratory viruses. All SARI cases and deaths are notified in the SIMI-SP database, regardless of hospitalisation.

We included all SARI related hospitalisations and deaths notified in São Paulo state between March 15 and August 29, 2020. Given that recent data is incomplete due to reporting delays^25^ and to avoid biases, we limited our analysis to patients with symptoms onset between these dates (epidemiological weeks 10 – 35). We also included SARI cases with unknown etiology, as those are likely related to COVID-19 but not lab-confirmed due to low rates of COVID-19 testing in Brazil^26^ and socioeconomic bias in testing.^23^

Zip code information was only available for cases reported in São Paulo state. Data were geocoded using the patient’s self-reported home address or postal code with Galileo (www.img.com.br) and Google API. For our analysis, we aggregated these data to the census tract level (n=68 296), the smallest administrative unit reported by the Brazilian census for the purpose of spatial statistical analysis. In the state of São Paulo, 95% of the census tracts have a population size between 136 and 1347 individuals (median=724). Information on the health facility where each case was notified was linked to the National Registry of Health Facilities (Cadastro Nacional de Estabelecimentos de Saúde), which includes information on the mode of healthcare provision (public and private).

The race of patients was partially self-declared and partially identified by a health professional. Race was categorised as either ‘White’, ‘Black’, ‘Asian’ (East or Southeast Asian), ‘*Pardo*’ (mixed ethnic ancestries with diverse skin colours)^27^ or Indigenous. Race information was missing for 53 480 (23.9%) of retrieved SARI cases, and was imputed using the racial distribution of the census tract of residence (see online supplemental materials for details). About 0.1% of the population in São Paulo self-identified as Indigenous^28^ and since only 166 Indigenous patients (0.07%) were recorded, they were not considered in our analysis.

10.1136/bmjgh-2021-004959.supp1 Supplementary data

#### Socioeconomic data

We obtained data on municipality-level socioeconomic factors from the latest population census (2010) compiled by the Brazilian Institute of Geography and Statistics (Instituto Brasileiro de Geografia e Estatística; IBGE). We selected indicators based on their relevance to the social determinants of health as defined by the WHO’s Commission on Social Determinants of Health,^29^ which includes income and income distribution, education, employment and job security, and access to healthcare. We included household income per capita, population density and income inequality (Gini Index). We also determined the proportion of residents with a primary education or lower, employment to population ratio and the proportion of the working population without a formal labour market contract or social security. The road network distance from the centroid of each census tract to the nearest healthcare facility was computed considering all 830 facilities that hospitalised patients with SARI via the public healthcare system (Sistema Único de Saúde; SUS). Information on employment status and comorbidities during the epidemic was retrieved from the National Household Sample Survey (Pesquisa Nacional por Amostra de Domicílios (PNAD) COVID-19), a national telephone survey conducted by IBGE with over 1 888 560 interviews between May and September 2020. Details are described in online supplemental materials.

#### Seroprevalence data

To assess the broader risk of SARS-CoV-2 infection beyond hospitalisation, we adopted seroprevalence data collected as part of the national Covid-IgG study from blood donors aged 16 – 69 living in São Paulo city.^30^ Given that samples were taken across the city, a population-weighted cluster sample of approximately 1000 blood donations were tested each month between February and August 2020 using a chemiluminescence assay that detects IgG against the SARS-CoV-2 nucleocapsid (N) protein (Abbott, Chicago, USA). Self-reported race and education level were recorded at the time of blood donation. To correct for differences in the age-sex distribution of blood donors compared with the population of São Paulo, we applied an age-sex normalisation to the measured prevalence. Details about the data collection methods can be found in Buss *et al.* ’s 30 study. 31

### Daily mobility and NPIs

To assess the ability of populations to self-isolate at the local level, we used daily mobile phone data provided by In-Loco (https://www.inloco.com.br/covid-19)^32^ for the greater metropolitan area of São Paulo (Região Metropolitana de São Paulo; RMSP). These data were aggregated using a hexagonal grid based on the global H3 index at resolution 8. Each cell has an edge of approximately 460 m and an area of 0.74 km^2^ (https://h3geo.org/docs/core-library/restable). For each H3 cell, the social isolation index was measured as the number of people who did not leave their cell of residence during the day, divided by the number of residents in that cell. Each mobile phone was assigned to an H3 cell based on the owner’s location of residence during the evening and their travel history. The racial composition and income level of each cell were determined using dasymetric interpolation (online supplemental materials).

We also used municipality and state-level data on NPIs from a continuous survey conducted between 13 May and 31 July 2020.^33^ The survey had 13 questions related to the implementation and easing of social distancing measures, and responses were obtained from 612 mayors in São Paulo (94.8% of the total).

### Data analysis

#### Probability of hospitalisation and death

We conducted an individual-level analysis to estimate the probability of reporting a SARI hospitalisation given a patient’s race and average income level in their census tract of residence. Census tracts were grouped by quantiles of income per capita into six categories as presented in the results. Similarly, we determined the probability of death from SARI given a patient’s race, income and administrative type of the health facility where the patient was hospitalised (public or private). Both probabilities were standardised by age and sex to account for demographic differences between groups. They were calculated for every month between March and August 2020. Probabilities for each age-sex group were estimated empirically using relative frequencies. ORs using White patients and the highest income level as reference groups were computed. CIs were calculated using bootstrapping. Details are in online supplemental materials.

#### Seroprevalence by socioeconomic status

We calculated the proportion of individuals by education and race category with detectable anti-SARS-CoV-2 antibodies between February and October 2020. 95% CIs were calculated by the exact binomial method and corrected for the specificity and sensitivity of the test.^31^

#### Socioeconomic drivers and hospitalisation risk

An ecological spatiotemporal regression analysis was conducted at the municipality level for São Paulo state (n=645 municipalities) to assess the monthly risk of hospitalisation and its association with socioeconomic factors between 1 March and 29 August 2020. To further understand the association between socioeconomic conditions and COVID-19 risk, we conducted the same analysis at the census tract level (n=30 815) for the RMSP, where the majority of cases were concentrated. The relative risk of hospitalisation was estimated using a hierarchical Bayesian model composed of a generalised log-linear model with spatially structured and unstructured random effects to account for spatial autocorrelation and time-varying random effect. The spatial structure is characterised by population movement between municipalities from 1 March to 15 August 2020 defined by In-Loco mobile geolocation data summarised elsewhere.^32^ A detailed description of the model and interpretation, covariates and diagnostics can be found in online supplemental materials.

#### Population response to NPIs

We used an event study design^34^ to examine how different socioeconomic groups changed their daily mobility levels in response to the introduction and relaxation of NPIs in the RMSP. We compared changes in mobility patterns of the population living in H3 cells with predominantly White versus predominantly Black or *Pardo* residents, as well as of the population living in areas of the wealthiest and poorest income quintiles. The daily isolation index from hexagons was regressed on a set of relative time dummies that indicated the number of days before and after the first NPI introduction in São Paulo state. Hexagon fixed effects controlled for unobserved time-invariant determinants of self-isolation while day fixed effects controlled for temporal shocks common to all hexagons. We further included an additional time-varying control variable, representing the number of days relative to the first confirmed SARI case in each hexagon, and a dummy variable indicating the period of NPI relaxation in each municipality. Sensitivity analyses were performed and discussed in online supplemental materials.

We employed a multinomial logistic regression to estimate the probability that employed individuals would be working face to face, teleworking, or taking paid or unpaid leave. Differences in the work status of individuals by race, education and occupation were calculated while controlling for age and sex. Thirty-five groups of employment occupations listed in the PNAD COVID-19 survey were aggregated to 10 SCO-08 one-digit occupational groups defined by the International Labour Organization. We further disaggregated health professionals and health technicians (online supplemental materials).

#### Comorbidities

Information on patient comorbidities was missing for approximately 61.6% of the cases in the SIMI-SP database. We estimated the incidence of comorbidities for the population of São Paulo state using the PNAD COVID-19 data. A binomial logistic regression was used to estimate the OR of having at least one comorbidity, by race and education attainment (preprimary, primary, secondary and tertiary), while controlling for age and sex for São Paulo state. The comorbidities considered were chronic obstructive pulmonary disease, diabetes, hypertension or cardiovascular disease such as myocardial infarction, angina or heart failure. CIs for the ORs were calculated taking into account PNAD’s complex sample design.

## Results

### SARIs capture COVID-19-related hospitalisations

**Figure 1:**
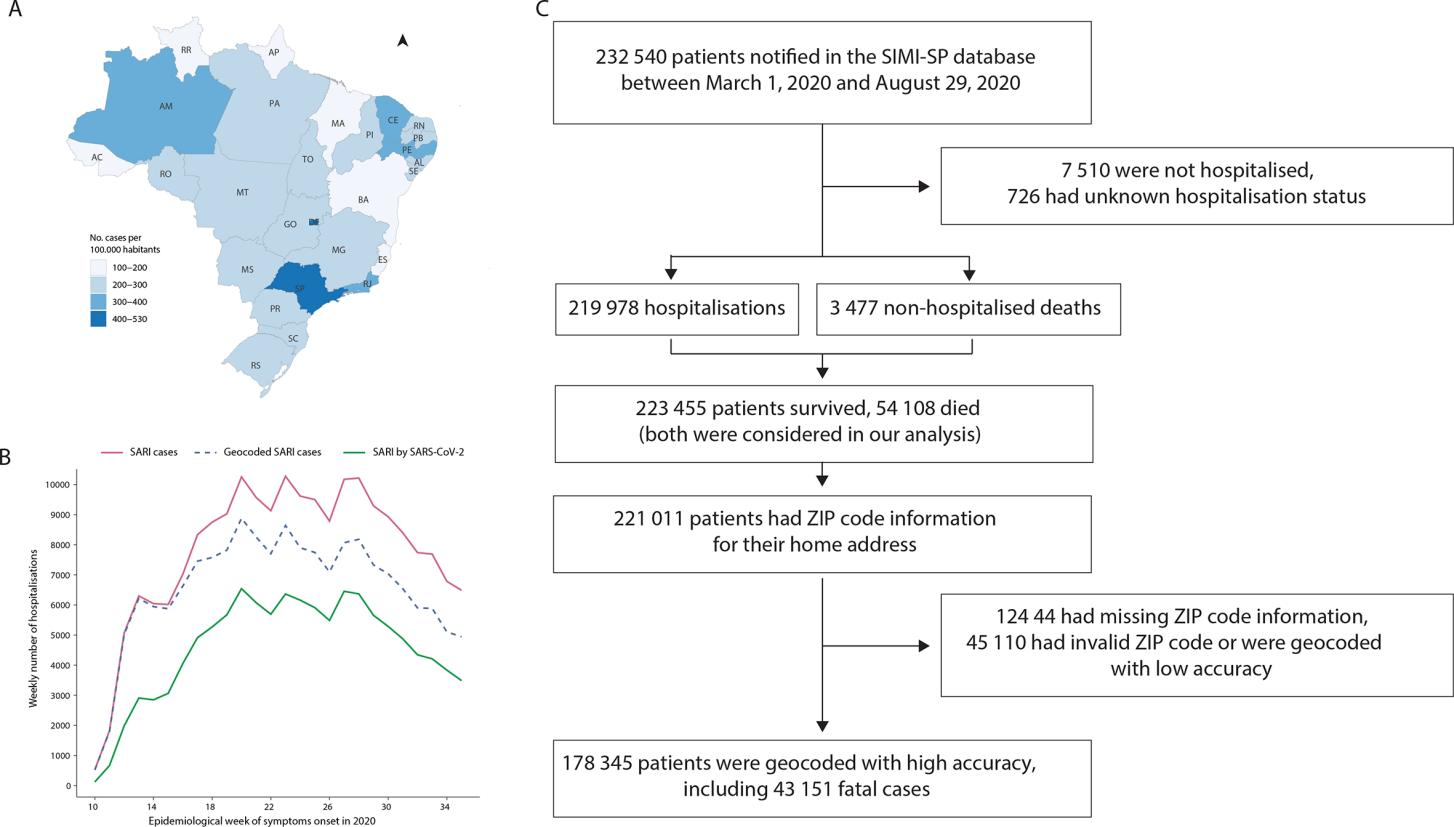
Severe acute respiratory infection (SARI) hospitalisations in São Paulo state. (A) Number of hospitalisations per 100 000 habitants by state in Brazil between 1 March and 29 August 2020. (B) Number of SARI hospitalisations for the state of São Paulo by week of symptom onset. (C) Flow chart of Sistema de Monitoramento Inteligente de São Paulo (SIMI-SP) data processing (Source: https://covid.saude.gov.br).

Between 1 March and 29 August 2020, São Paulo state had the highest number of SARI hospitalisations per 100 000 habitants compared with all states in Brazil (figure 1A). A time series illustrating the number of SARI hospitalisations is presented in figure 1B. During this time, 232 540 patients were notified in the SIMI-SP database (figure 1C), from which 127 434 (54.8%) had a confirmed COVID-19 diagnosis and 103 360 (44.4%) were diagnosed with SARI of unknown or missing aetiology. From these, 223 455 were hospitalised (98.4%) or died without hospitalisation (1.6%). From the non-hospitalised cases, we only selected deaths; 54 108 patients died, of which 52.5% were White, 20.2% were *Pardo*, 6.13% were Black, 1.96% were Asian, 0.052% were Indigenous and 19.6% did not have race information. We geocoded 178 345 (79.8%) of all SARI cases considered in our analyses with high accuracy at either street address, route or neighbourhood level without compromising personal privacy.

### Individual risk to hospitalisation and death varies by race, socioeconomic status and hospital type

**Figure 2:**
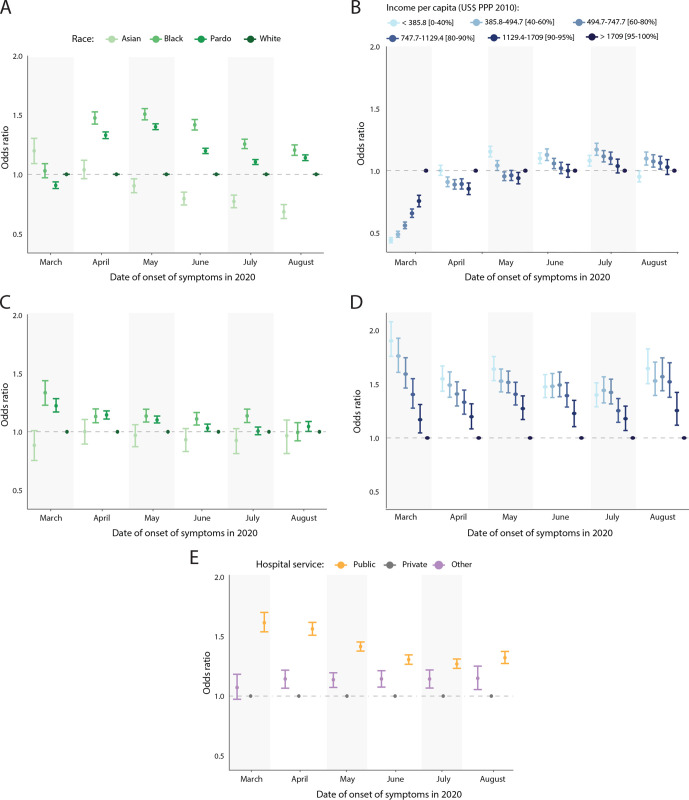
Individual-level hospitalisation and death risk by age-standardised OR. (A) OR for severe acute respiratory infection (SARI) hospitalisation by race. (B) OR for SARI hospitalisation by income. (C) OR for death among patients with SARI by race. (D) OR for death among patients with SARI by income. (E) OR for death among patients with SARI by hospital type. PPP, purchasing power parity.

During the first month of the COVID-19 epidemic (March) in Brazil, hospitalised patients were more likely to be White or Asian and come from census tracts with higher income per capita (figure 2A, B). During this period, people living in low-income areas were less likely (OR: 0.44, 95% CI 0.42 to 0.46) to be hospitalised with SARI compared with high-income areas. We found that point estimates of inequality levels do not change substantially when observations with missing race information are dropped, though CIs become smaller (online supplemental figure S1). This coincides with the early introduction of COVID-19 in Brazil, when the first infections occurred among higher income travellers returning from overseas.^20 23^

As the epidemic progressed from April onwards, patients were on average more likely to be from low-income census tracts (April to July, OR: 1.08, 95% CI 1.04 to 1.12), except for August, when patients were less likely to be from low-income census tracts (OR: 0.95, 95% CI 0.91 to 1.00). Similarly during this time period, Black Brazilians and *Pardos* became more likely to be hospitalised with SARI than Whites (OR: 1.41, 95% CI 1.37 to 1.46; OR: 1.26, 95% CI 1.23 to 1.28, respectively), while Asians became the least likely to be hospitalised (OR: 0.88, 95% CI 0.82 to 0.94). These results were further confirmed by our seroprevalence findings, where both crude and adjusted prevalence (for age, sex, sensitivity and specificity of the sex) showed that anti-SARS-CoV-2 antibodies were highest in Black blood donors and those with low educational attainment across all age groups (online supplemental figure S2).

Once hospitalised, Black and *Pardo* patients were more likely to die from SARI than White patients between March and August (OR: 1.14, 95% CI 1.07 to 1.21; 1.09, 95% CI 1.05 to 1.13, respectively) (figure 2C). This difference was more pronounced in March and decreased over time. Because our analysis does not control for comorbidities, these results indirectly reflect the differences in the incidence of comorbidities across racial groups. We found that patients living in the poorest census tracts were more likely to die from SARI compared with patients from the wealthiest tracts (OR: 1.60, 95% CI 1.48 to 1.74) (figure 2D). Likewise, patients treated in public hospitals were more likely to die than patients treated in private hospitals throughout the epidemic (OR: 1.40%, 95% CI 1.34% to 1.46%) (figure 2E). Racial differences in the probability of death decreased when considering only patients hospitalised at public health facilities but persisted among patients in private facilities (online supplemental figure S3).

### Geographic variation in hospitalisation risk is driven by mobility and socioeconomic status

**Figure 3:**
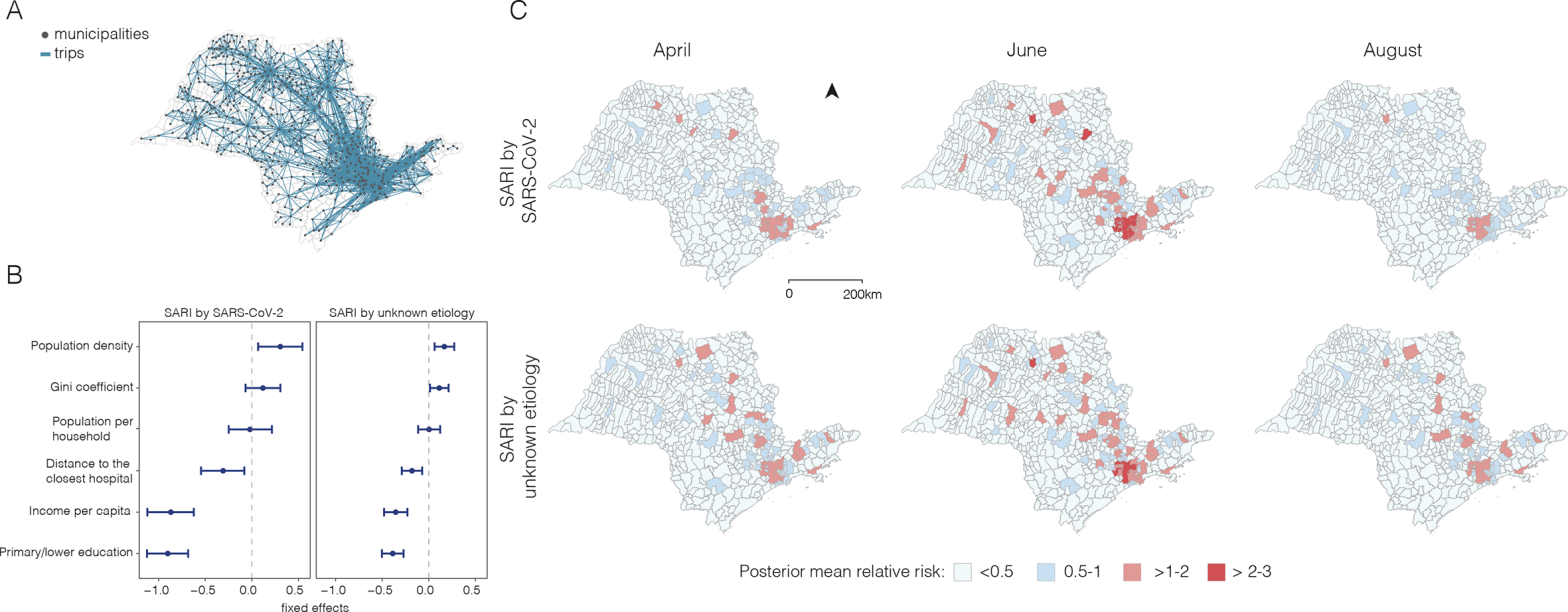
Hospitalisation risk by municipality in São Paulo state. (A) Human movement between municipalities based on In-Loco mobile phone data retrieved from March to August 2020. (B) Fixed effects and 95% credible intervals for socioeconomic covariates. (C) Relative risk of severe acute respiratory infection (SARI) hospitalisation at the municipality level.

To understand the geographical variation in SARI hospitalisation, we estimated and mapped the relative risk of SARI hospitalisation at the municipality level (n=645 municipalities) for São Paulo state by month using a model with a spatial structure defined by human movement fluxes derived from anonymised mobile phone data (figure 3A) and covariates related to socioeconomic status (figure 3B). Overall, municipalities with higher levels of movement exchange with the RMSP had higher monthly risk of SARI hospitalisation (figure 3C). We found a lower risk of SARI by SARS-CoV-2 hospitalisation in municipalities with high income per capita (fixed effect=−0.87, 95% CI −1.12 to −0.62) and high proportion of adult residents with a primary education or lower (−0.90, 95% CI −1.12 to −0.68). Municipalities with fewer nearby public health facilities were also found to have lower risk of hospitalisation (−0.31, 95% CI −0.55 to −0.08). We also found a higher risk of SARI hospitalisation in municipalities with higher population density (0.31, 95% credible interval: 0.07–0.54).

**Figure 4:**
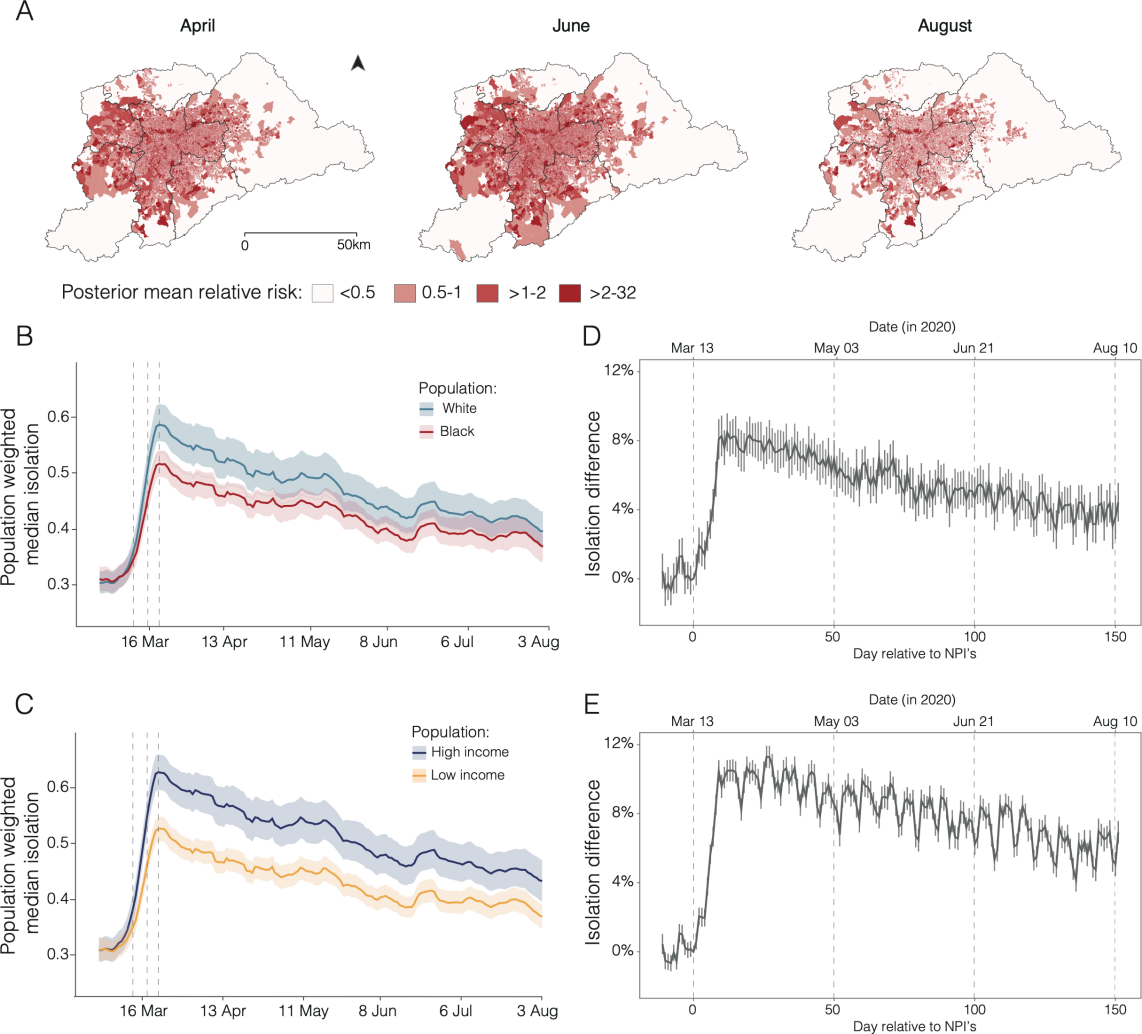
Differential risk based on varying ability to self-isolate in the Região Metropolitana de São Paulo (RMSP). (A) Relative risk of severe acute respiratory infection (SARI) hospitalisation for the RMSP. (B) Seven-day moving average of daily isolation levels by race. (C) Seven-day moving average of daily isolation levels by income. (D) Difference in daily social isolation by race after the introduction of non-pharmaceutical intervention (NPI). (E) Difference in daily social isolation by income after the introduction of NPIs. In panels (B) and (C), solid lines show population-weighted median isolation levels and shaded areas show population-weighted IQR (25%–75%). Dashed vertical lines indicate the dates of NPIs that enabled school closure (13 March was the state NPI) and non-essential activities (18 and 22 March, municipal and state NPIs, respectively).

We found that over time, the risk of SARI hospitalisation increased particularly in municipalities near and within the RMSP (greater metropolitan area of São Paulo), where 70% of the SARI cases reported for the state are concentrated (figure 4A). By mapping risk at the census tract level (n=30 815) for the RMSP, we detected increasing risk starting from São Paulo city (central region). By June, almost all of the census tracts in and near the city centre were found to have high relative risk, but this risk decreased by August.

### Lower ability to self-isolate by disadvantaged groups

Differential risk to SARI in the RMSP was also associated with daily mobility levels. Before the implementation of NPIs on 13 March, mobility levels were similar across all socioeconomic groups (figure 4B, C). However, 14 days after the introduction of NPIs, isolation levels were 8.2% (95% CI 7.2% to 9.2%) higher in predominantly White areas compared with predominantly Black areas. Similarly, 27 days after the introduction of state-level NPIs, isolation levels were 11.2% (95% CI 10.6% to 11.9%) higher in the wealthiest than in the poorest areas. Overall, we detected a decreasing trend in isolation levels over time, and the magnitude of the differences in social isolation levels between areas with predominantly White and Black populations decreased to only 4.4% (95% CI 3.3% to 5.5%) 151 days after the introduction of the NPIs (figure 4D).

**Figure 5:**
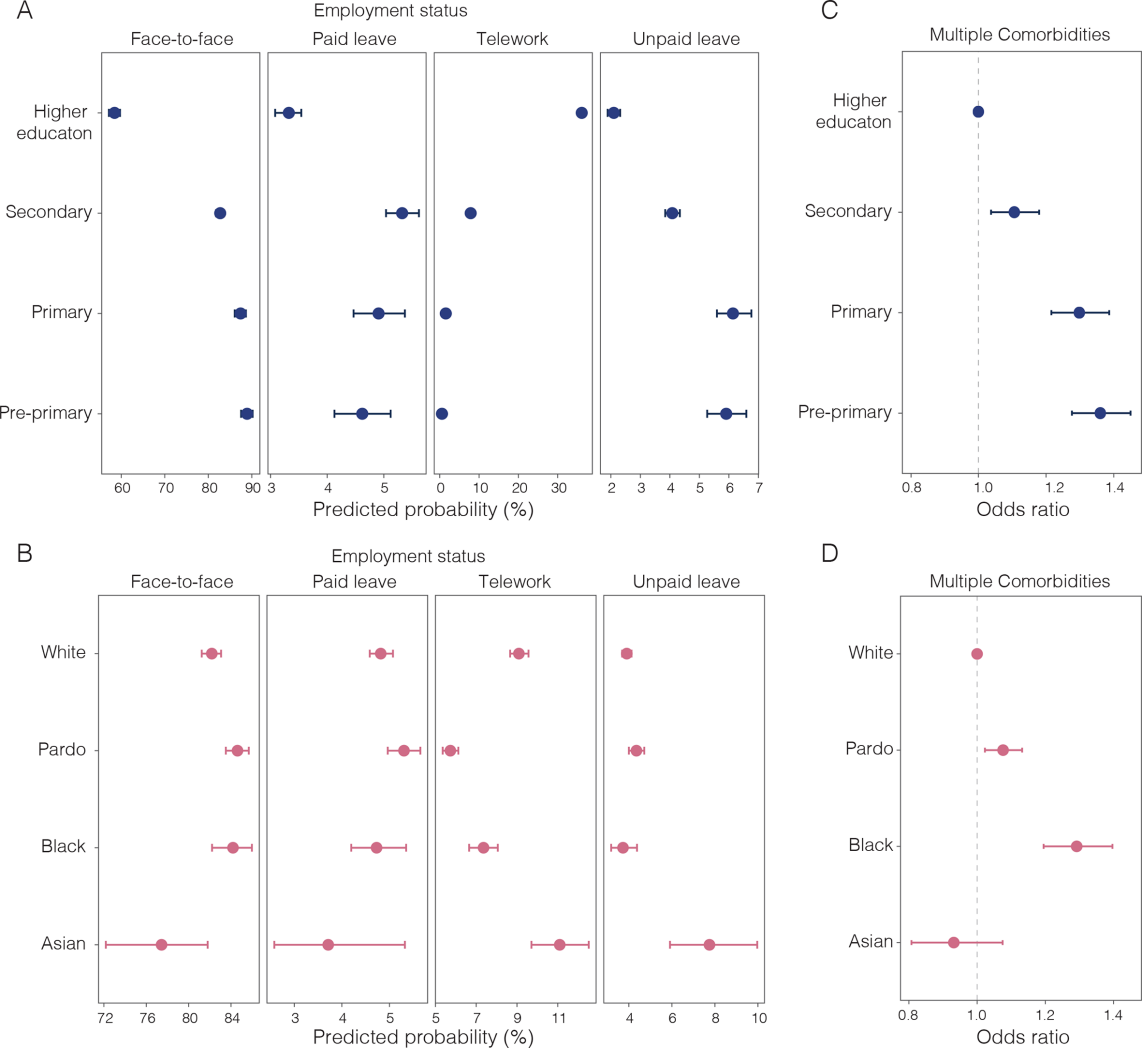
Inequalities in working conditions and comorbidities. (A) Probability of different working conditions by education attainment. (B) Probability of different working conditions by race. (C) OR (OR=1) of having one or more comorbidities by education attainment. (D) OR (OR=1) of having one or more comorbidities by race. Comorbidities considered include chronic obstructive pulmonary disease (COPD), diabetes, hypertension or cardiovascular disease such as infarction, angina and heart failure. Horizontal lines show 95% CIs (Source: Pesquisa Nacional por Amostra de Domicílios (PNAD) COVID-19/Instituto Brasileiro de Geografia e Estatística (IBGE),^17^ July to September 2020).

Finally, we investigated the differential risk to SARI based on workers’ position in the labour market using data from the PNAD COVID-19 survey. After the introduction of NPIs, workers employed in low-skilled jobs or essential services were more likely to keep working face to face than workers in professional or managerial positions (online supplemental figure S4). Workers with pre-primary education were more likely to work in occupations that require in-person contact than workers with tertiary education (probability (PR): 0.89, 95% CI 0.87 to 0.90 compared with PR: 0.58, 95% CI 0.57 to 0.60) and less likely to work in occupations that allow teleworking (PR: 0.005, 95% CI 0.004 to 0.007 vs PR: 0.36, 95% CI 0.35 to 0.37, respectively) (figure 5A). When controlling for education and formal or informal employment, we found no substantial difference between racial groups in the probability of working face to face or teleworking (figure 5B). Nonetheless, because Black and Pardo populations are disproportionally employed in informal and low-skilled jobs, these racial groups were, in general, more likely to be working face to face during our study period.

### Disadvantaged groups have more comorbidities

We found that population groups at risk of death from SARI were also more likely to have comorbidities known to aggravate COVID-19 severity. Compared with the population with tertiary education in São Paulo state, individuals with primary education or lower are more likely to have one or more comorbidities (OR: 1.36, 95% CI 1.27 to 1.45) (figure 5C). Similarly, Black individuals were also more likely to have one or more comorbidities than White individuals (OR: 1.29, 95% CI 1.19 to 1.39) (figure 5D). OR estimates for each health condition are summarised in online supplemental figure S5.

## Discussion

Our study shows that socially disadvantaged groups are disproportionately more likely to be hospitalised and die from SARI. We find that the differential health outcomes can be explained by structural inequities linked to the incidence of comorbidities and to socioeconomic conditions, which limit the ability of low-income and non-White populations to socially isolate and reduce their access to quality health services.

Social and racial inequalities shape the risk of SARI hospitalisation and death. After the initial phase of international imports in Brazil,^23^ Black or *Pardo* Brazilians and individuals residing in low-income areas were more likely to be hospitalised and die with SARI compared with White individuals and those from wealthier areas, which aligns with recent findings.^17 18^ While these results report of severe cases of infection, we find similar results when looking broadly at the population level of COVID-19 infection. Our assessment of anti-SARS-CoV-2 antibodies in blood donors categorised by demographic background further confirms that Black Brazilians and those with lower socioeconomic status are disproportionately exposed to COVID-19.

Patients hospitalised in public health facilities were more likely to die than those in private health facilities. The uneven access to health services explains some but not all the inequality in the risk of death from COVID-19, since racial inequalities in death probability persist among patients within private hospitals. Potential factors influencing this inequality include higher comorbidities among poor Black patients and the lower access to private care among low-income individuals who are disproportionately Black. Other important factors include the disadvantage of having multiple comorbidities, which are more prevalent among Blacks and *Pardos* and those with lower education.

We found that hospitalisation risk is higher for populations living in municipalities that travel to and from the RMSP, with low income per capita and high population density compared with the rest of São Paulo state. These populations mainly reside in the RMSP, which contains nearly half of the population in São Paulo state and where bias in testing is evident in regions of lower socioeconomic status^23^ (online supplemental figure S6). The risk of SARI hospitalisation is particularly elevated in São Paulo city, where seroprevalence estimates from blood donors show that anti-SARS-CoV-2 antibodies were highest in older Black Brazilians and those with lower educational attainment.

We show that inequalities in the risk of SARI hospitalisation were partially explained by differential mobility responses to social distancing guidelines, similar to the UA.^35^ In wealthier and predominantly White neighbourhoods, people were able to isolate more, faster, and sustain isolation for long periods of time. We also found that occupational factors played a key role in influencing individuals’ ability to physically isolate. Among the working population, low-income and Black workers were less likely to receive a furlough from work or telework. Due to systemic inequalities in education and the labour market, these groups are disproportionately employed in precarious job positions with no social security and dependent on day-to-day income,^36^ limiting their ability to socially distance through telework and reductions in daily mobility. The lack of capacity to self-isolate and curtail mobility in these occupations may increase exposure and facilitate SARS-CoV-2 transmission.

Our study has limitations that may have underestimated the level of inequality. First, geocoding cases may have discarded patients from poor census tracts where accuracy is limited.^37^ Second, using data aggregated for various administrative levels has inherent limitations due to ecological fallacy and the modifiable areal unit problem.^38^ Finally, the 2010 Brazilian population census and PNAD COVID-19 survey may have limited the capture of socioeconomic changes in the last decade and inclusion of extremely wealthy individuals.^39^ Additionally, disadvantaged groups can be under-represented in health administrative records because of their lower access to healthcare. Given that São Paulo is the wealthiest state and has the most robust healthcare system in Brazil,^40^ it is possible that the impact of inequalities is more severe in other states.

Our findings on the difference of SARI death risk reveal stark inequalities in access to healthcare. Health is a constitutional right and a state responsibility in Brazil.^41^ The country’s public healthcare system (SUS) is designed to provide universal health coverage without out-of-pocket costs, and regionally planned to improve spatial coverage of services. Yet, previous studies have found that access to COVID-19 health services tends to be lower in less developed regions in the country,^41 42^ particularly among low-income and black communities.^43^ Only 25% of Brazilians have access to private healthcare via health insurance, reflecting how inequality in access to quality healthcare is largely driven by income.^44^ This leaves 75% of the population solely reliant on a chronically underfunded public healthcare system, which highlights a double disadvantage for low-income and non-White populations, who are more likely to be infected and deprived of care. Strengthening healthcare access and its capacity will be critical for reducing health inequities during this and forthcoming public health emergencies.^42^

Our findings on socioeconomic risk factors could help guide vaccine allocation in diverse settings to achieve equitable access. Ensuring that disadvantaged groups, especially those that have in-person occupations and live in crowded and deprived areas, receive vaccination will help prevent and slow down community transmission. While race is not a risk factor in itself, it is critical to consider systemic inequalities that lead Black and *Pardo* communities to be over-represented among low socioeconomic groups, to have higher rates of severe COVID-19 infection, and comorbidities that exacerbate their risk of death. Therefore, including disadvantaged populations among priority groups for vaccination could help reduce health inequities instead of exacerbating them.^45^

As shown in our study, the combination of social, racial and health inequalities in fostering high mortality risk among systematically marginalised communities exemplifies the syndemic nature of the COVID-19 pandemic.^3 46^ The negative impact of the COVID-19 pandemic on population health is driven by the accumulation and interaction of two or more adverse conditions, often influenced by the social determinants of health.^47^ Therefore, in order to reduce inequalities in COVID-19-related health risk, action is needed to address all factors that contribute to health inequalities. Prior to the start of the pandemic, these determinants have already shaped pre-existing living conditions of disadvantaged groups—such as poor education, precarious work, residential segregation and inadequate housing—which disproportionately impact their access to quality healthcare and expose them to the onset of comorbidities.^47 48^ In order to reduce inequalities in COVID-19-related health risk, it is essential to understand what made them vulnerable in the first place by addressing the adverse social determinants that shape an individual’s life course. While a key role is played by healthcare systems, action is also required from other stakeholders. COVID-19 control measures affect people differently based on varying levels of financial resources and support available to them, thus governments and industries must work together to address these inequalities by mobilising resources and tools^49^ and by developing targeted interventions at both national^50^ and local levels.^51^

Our study highlights the need for additional research to comprehend the effects of social and health inequalities during pandemics. First, an assessment of the inequality in access to quality care within public and private health facilities and its risk factors is needed to better understand mortality in different hospital settings. Second, our study shows that population response to NPIs can vary significantly based on social circumstances, suggesting that future studies should also consider socioeconomic aspects when evaluating the effectiveness of NPIs. Third, more data are needed on whether social safety net programmes that are guaranteeing income for disadvantaged groups during the pandemic (eg, Brazil’s emergency cash payment) may have enabled people to reduce their mobility. Nevertheless, our study has shown the impact of social inequities on COVID-19 hospitalisation and death, thus informing future research and policies related to the health impacts of COVID-19 in Latin America.

## Data Availability

Data are available upon request. The data sets used and/or analysed during the current study are available from the corresponding authors on reasonable request.

## References

[1] Ahmed F, Ahmed Na'eem, Pissarides C, et al. Why inequality could spread COVID-19. Lancet Public Health 2020;5:e240. 10.1016/S2468-2667(20)30085-2 32247329 PMC7270465

[2] Walker PGT, Whittaker C, Watson OJ, et al. The impact of COVID-19 and strategies for mitigation and suppression in low- and middle-income countries. Science 2020;369:413–22. 10.1126/science.abc0035 32532802 PMC7292504

[3] Horton R. Offline: COVID-19 is not a pandemic. Lancet 2020;396:874. 10.1016/S0140-6736(20)32000-6 32979964 PMC7515561

[4] Bambra C, Riordan R, Ford J, et al. The COVID-19 pandemic and health inequalities. J Epidemiol Community Health 2020;74:964–8. 10.1136/jech-2020-214401 32535550 PMC7298201

[5] Marmot M, Wilkinson R. Social determinants of health. OUP Oxford, 2005.

[6] Bartley M. Health inequality: an introduction to concepts, theories and methods. John Wiley & Sons, 2016.

[7] Dubey S, Biswas P, Ghosh R, et al. Psychosocial impact of COVID-19. Diabetes Metab Syndr 2020;14:779–88. 10.1016/j.dsx.2020.05.035 32526627 PMC7255207

[8] Emeruwa UN, Ona S, Shaman JL, et al. Associations between built environment, neighborhood socioeconomic status, and SARS-CoV-2 infection among pregnant women in New York City. JAMA 2020;324:390–2. 10.1001/jama.2020.11370 32556085 PMC7303894

[9] Chen JT, Krieger N. Revealing the unequal burden of COVID-19 by income, Race/Ethnicity, and household crowding: US County versus ZIP code analyses. J Public Health Manag Pract 2021;27 Suppl 1, COVID-19 and Public Health: Looking Back, Moving Forward:S43–56. 10.1097/PHH.0000000000001263 32956299

[10] Abedi V, Olulana O, Avula V. Racial, economic, and health inequality and COVID-19 infection in the United States. J Racial Ethn Health Disparities 2020:1–11. 10.1007/s40615-020-00833-4 PMC7462354 32875535

[11] UK Office for National Statistics. Coronavirus (COVID-19) related deaths by ethnic group, England and Wales - Office for National Statistics, 2020. Available: https://www.ons.gov.uk/peoplepopulationandcommunity/birthsdeathsandmarriages/deaths/articles/coronavirusrelateddeathsbyethnicgroupenglandandwales/2march2020to10april2020 [Accessed 20 Nov 2020].

[12] Niedzwiedz CL, O'Donnell CA, Jani BD, et al. Ethnic and socioeconomic differences in SARS-CoV-2 infection: prospective cohort study using UK Biobank. BMC Med 2020;18:160. 10.1186/s12916-020-01640-8 32466757 PMC7255908

[13] Drefahl S, Wallace M, Mussino E, et al. A population-based cohort study of socio-demographic risk factors for COVID-19 deaths in Sweden. Nat Commun 2020;11:5097. 10.1038/s41467-020-18926-3 33037218 PMC7547672

[14] Dragano N, Rupprecht CJ, Dortmann O. Higher risk of COVID-19 hospitalization for unemployed: an analysis of 1,298,416 health insured individuals in Germany. medRxiv 2020:2020.06.17.20133918. 10.1007/s00103-021-03280-6 PMC7841971 33507323

[15] Argoty-Pantoja AD, Robles-Rivera K, Rivera-Paredez B, et al. COVID-19 fatality in Mexico's Indigenous populations. Public Health 2021;193:69–75. 10.1016/j.puhe.2021.01.023 33743216 PMC7877204

[16] Pan D, Sze S, Minhas JS, et al. The impact of ethnicity on clinical outcomes in COVID-19: a systematic review. EClinicalMedicine 2020;23:100404. 10.1016/j.eclinm.2020.100404 32632416 PMC7267805

[17] Baqui P, Bica I, Marra V, et al. Ethnic and regional variations in hospital mortality from COVID-19 in Brazil: a cross-sectional observational study. Lancet Glob Health 2020;8:e1018–26. 10.1016/S2214-109X(20)30285-0 32622400 PMC7332269

[18] Ribeiro KB, Ribeiro AF, de Sousa Mascena Veras MA, et al. Social inequalities and COVID-19 mortality in the city of São Paulo, Brazil. Int J Epidemiol 2021:dyab022. 10.1093/ije/dyab022 33657223 PMC7989375

[19] Facundo A, Chancel L, Thomas P. World inequality report 2018, 2017.

[20] Jesus JGde, Sacchi C, Candido DdaS, et al. Importation and early local transmission of COVID-19 in Brazil, 2020. Rev Inst Med Trop Sao Paulo 2020;62:e30. 10.1590/s1678-9946202062030 32401959 PMC7232955

[21] Henriques CMP, Vasconcelos W, et al. Crises dentro dA crise: respostas, incertezas E desencontros no combate à pandemia dA Covid-19 no Brasil. Estud Av 2020;34:25–44. 10.1590/s0103-4014.2020.3499.003

[22] Rezende LFM, Thome B, Schveitzer MC, et al. Adults at high-risk of severe coronavirus disease-2019 (Covid-19) in Brazil. Rev Saude Publica 2020;54:50. 10.11606/s1518-8787.2020054002596 32491091 PMC7234208

[23] de Souza WM, Buss LF, Candido DdaS, et al. Epidemiological and clinical characteristics of the COVID-19 epidemic in Brazil. Nat Hum Behav 2020;4:856–65. 10.1038/s41562-020-0928-4 32737472

[24] Secretaria da saúde do estado de São Paulo. SIMI-SP: Pacientes internados POR Síndrome Respiratória Aguda Grave (SRAG). Available: https://www.saopaulo.sp.gov.br/planosp/simi/ [Accessed 25 Oct 2020].

[25] Niquini RP, Lana RM, Pacheco AG, et al. Srag POR COVID-19 no Brasil: descrição E comparação de características demográficas E comorbidades CoM SRAG POR influenza E CoM a população geral. Cad. Saúde Pública 2020;36:e00149420. 10.1590/0102-311x00149420 32725087

[26] Hasell J, Mathieu E, Beltekian D, et al. A cross-country database of COVID-19 testing. Sci Data 2020;7:345. 10.1038/s41597-020-00688-8 33033256 PMC7545176

[27] Telles E, Paschel T, Black WI. Who is black, white, or mixed race? how skin color, status, and nation shape racial classification in Latin America. Am J Sociol 2014;120:864–907. 10.1086/679252 25848671

[28] IBGE - Instituto Brasileiro de Geografia e Estatística. Pesquisa Nacional por Amostra de Domicílios Contínua (PNAD) COVID-19. Microdados, 2020.

[29] Marmot M, Friel S, Bell R, et al. Closing the gap in a generation: health equity through action on the social determinants of health. The Lancet 2008;372:1661–9. 10.1016/S0140-6736(08)61690-6 18994664

[30] Buss LF, Prete CA, Abrahim CMM, et al. Three-quarters attack rate of SARS-CoV-2 in the Brazilian Amazon during a largely unmitigated epidemic. Science 2021;371:288–92. 10.1126/science.abe9728 33293339 PMC7857406

[31] Diggle PJ. Estimating prevalence using an imperfect test. Epidemiol Res Int 2011;2011:1–5. 10.1155/2011/608719

[32] Peixoto PS, Marcondes D, Peixoto C, et al. Modeling future spread of infections via mobile geolocation data and population dynamics. An application to COVID-19 in Brazil. PLoS One 2020;15:e0235732. 10.1371/journal.pone.0235732 32673323 PMC7365450

[33] de Souza Santos AA, Candido DdaS, de Souza WM, et al. Dataset on SARS-CoV-2 non-pharmaceutical interventions in Brazilian municipalities. Sci Data 2021;8:73. 10.1038/s41597-021-00859-1 33664243 PMC7933188

[34] Weill JA, Stigler M, Deschenes O, et al. Social distancing responses to COVID-19 emergency declarations strongly differentiated by income. Proc Natl Acad Sci U S A 2020;117:19658–60. 10.1073/pnas.2009412117 32727905 PMC7443940

[35] Chang S, Pierson E, Koh PW, et al. Mobility network models of COVID-19 explain inequities and inform reopening. Nature 2021;589:82–7. 10.1038/s41586-020-2923-3 33171481

[36] Lustig N, Pabon VM, Sanz F. The impact of COVID-19 Lockdowns and expanded social assistance on inequality, poverty and mobility in Argentina, Brazil, Colombia and Mexico. ECINEQ, Society for the study of economic inequality, 2020. Available: https://ideas.repec.org/p/inq/inqwps/ecineq2020-558.html [Accessed 20 Nov 2020].

[37] Giest S, Samuels A. ‘For good measure’: data gaps in a big data world. Policy Sci 2020;53:559–69. 10.1007/s11077-020-09384-1

[38] Duranton G, Overman HG. Testing for localization using Micro-Geographic data. Rev Econ Stud 2005;72:1077–106. 10.1111/0034-6527.00362

[39] de SP. A distribuição de renda nas pesquisas domiciliares brasileiras: harmonização E comparação entre Censos, PNADs E POFs. Rev Bras Estud Popul 2015;32:165–88.

[40] Paim J, Travassos C, Almeida C, et al. The Brazilian health system: history, advances, and challenges. The Lancet 2011;377:1778–97. 10.1016/S0140-6736(11)60054-8 21561655

[41] Castro MC, Massuda A, Almeida G, et al. Brazil’s unified health system: the first 30 years and prospects for the future. The Lancet 2019;394:345–56. 10.1016/S0140-6736(19)31243-7 31303318

[42] Castro MC, de CLR, Chin T. Demand for hospitalization services for COVID-19 patients in Brazil. medRxiv 2020:2020.03.30.20047662.

[43] Pereira RHM, Braga CKV, Servo LM, et al. Geographic access to COVID-19 healthcare in Brazil using a balanced float catchment area approach. Soc Sci Med 2021;273:113773. 10.1016/j.socscimed.2021.113773 33609968 PMC7879934

[44] Matijasevich A, Russo G. Covid-19 in Brazil has exposed socio-economic inequalities and underfunding of its public health system. BMJ 2020 https://blogs.bmj.com/bmj/2020/06/19/covid-19-in-brazil-has-exposed-deeply-rooted-socio-economic-inequalities-and-chronic-underfunding-of-its-public-health-system/

[45] Emanuel EJ, Persad G, Kern A, et al. An ethical framework for global vaccine allocation. Science 2020;369:1309–12. 10.1126/science.abe2803 32883884 PMC8691258

[46] Singer M, Bulled N, Ostrach B, et al. Syndemics and the biosocial conception of health. Lancet 2017;389:941–50. 10.1016/S0140-6736(17)30003-X 28271845

[47] Abrams EM, Szefler SJ. COVID-19 and the impact of social determinants of health. Lancet Respir Med 2020;8:659–61. 10.1016/S2213-2600(20)30234-4 32437646 PMC7234789

[48] Barber S, Diez Roux AV, Cardoso L, et al. At the intersection of place, race, and health in Brazil: residential segregation and cardio-metabolic risk factors in the Brazilian longitudinal study of adult health (ELSA-Brasil). Soc Sci Med 2018;199:67–76. 10.1016/j.socscimed.2017.05.047 28821371

[49] Ismail SJ, Tunis MC, Zhao L, et al. Navigating inequities: a roadmap out of the pandemic. BMJ Glob Health 2021;6:e004087. 10.1136/bmjgh-2020-004087 PMC7825252 33479019

[50] Shadmi E, Chen Y, Dourado I, et al. Health equity and COVID-19: global perspectives. Int J Equity Health 2020;19:104. 10.1186/s12939-020-01218-z 32586388 PMC7316580

[51] Berkowitz RL, Gao X, Michaels EK, et al. Structurally vulnerable neighbourhood environments and racial/ethnic COVID-19 inequities. Cities Health 2020;31:1–4. 10.1080/23748834.2020.1792069 PMC9216191 35747269

